# Advances in Therapeutic Implications of Inorganic Drug Delivery Nano-Platforms for Cancer

**DOI:** 10.3390/ijms20040965

**Published:** 2019-02-22

**Authors:** Safia Naz, Muhammad Shamoon, Rui Wang, Li Zhang, Juan Zhou, Jinghua Chen

**Affiliations:** 1 Key Laboratory of Carbohydrate Chemistry and Biotechnology, Ministry of Education, School of Pharmaceutical Sciences, Jiangnan University, Wuxi 214122, China; safianaz26@hotmail.com (S.N.); jiangnanwangrui@163.com (R.W.); 6181504017@stu.jiangnan.edu.cn (L.Z.); 2 Medical School, The Australian National University, Canberra ACT 2600, Australia; shamoon1096@gmail.com

**Keywords:** cancer, drug delivery, inorganic agents, nano-carriers, therapeutics

## Abstract

Numerous nanoparticles drug delivery systems for therapeutic implications in cancer treatment are in preclinical development as conventional chemotherapy has several drawbacks. A chemotherapeutic approach requires high doses of chemotherapeutic agents with low bioavailability, non-specific targeting, and above all, development of multiple drug resistance. In recent years, inorganic nano-drug delivery platforms (NDDPs; with a metal core) have emerged as potential chemotherapeutic systems in oncology. One of the major goals of developing inorganic NDDPs is to effectively address the targeted anti-cancer drug(s) delivery related problems by carrying the therapeutic agents to desired tumors sites. In this current review, we delve into summarizing the recent developments in targeted release of anti-cancer drugs loaded in inorganic NDDPs such as mesoporous silica nanoparticles, carbon nanotubes, layered double hydroxides, superparamagnetic iron oxide nanoparticles and calcium phosphate nanoparticles together with highlighting their therapeutic performance at tumor sites.

## 1. Introduction

Cancer, being uncontrolled growth of malignant cells and invasive in nature, remains a worldwide threat to public health, which negatively affects the quality of life. In the USA, 1,762,450 new cancer cases were reported in 2019, and 606,880 deaths are projected to occur in 2019 [1]. The global trend is also increasing alarmingly as all types of cancer cases are expected to reach 22.2 million by 2030 [2]. Consistent with this, a huge upsurge in the global market of cancer drugs ($150 billion) is anticipated at an annual growth rate of 7.5–10.5% [3]. Hence, it is critical to effectively deliver highly potent active agents (drugs) to target tumor sites. This quest has driven the innovation in development of promising nano-drug delivery platforms (NDDPs) and fueled the advancements in the field of nanomedicine for cancer. Graphene, gold, selenium, silver, iron, and phosphorus containing NDDPs have experimentally yielded promising outcomes in various preclinical (i.e., in vitro and in vivo) models of cancer (Table 1). However, delivering anti-cancer agents to an intracellular target site is a long trip full of challenges such as the systemic circulation, accumulation of the drug in tumor lesions, reaching deep tumor penetration, uptake by tumor cells, intracellular disposition of drugs, and any unconquered stage may result in limited therapeutic efficiency [4,5].

Current cancer treatment options include surgical intervention, chemotherapy and radiation therapy or a combination of these options. These approaches are mainly nonselective which could also damage the healthy tissues. Moreover, conventional drug administration often requires high dosage or repeated administration to stimulate a therapeutic effect which can lower the overall efficacy and patient compliance resulting in severe side effects (i.e., toxicity) [5,21]. Thus, an optimized cancer therapy capable of delivering the right type of therapeutic agent to the right target tumorous site is warranted for an efficient localized control of the disease with minimal systemic toxicity. Research in recent decades has focused on controlled drug delivery systems [11,22,23,24] which have demonstrated the potential to address the challenges associated with conventional chemotherapeutic approach by improving treatment efficacy while avoiding toxicity in normal cells (highly selective accumulation in tumors).

**Figure 1:**
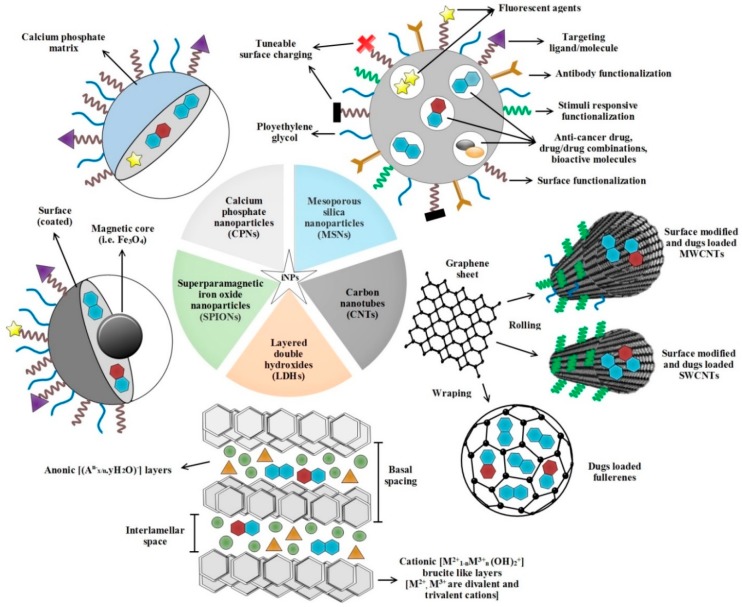
Schematic diagram depicting various inorganic NDDPs loaded with anti-cancer chemotherapeutic agents employed for cancer treatment. iNPs: inorganic nanoparticles; MWCNTs: multi-walled carbon nanotubes, SWCNTs: single-walled carbon nanotubes.

Anti-cancer drugs loaded in inorganic NDDPs (Figure 1) readily achieve prolonged systemic circulation (protection from degradation in the bloodstream), increased accumulation in tumor via enhanced permeability and retention (EPR) effect, enhanced drug stability and controlled drug release (improved cellular uptake) in tumor cells [25]. These inorganic NDDPs can control how the drugs are available to cancer cells and tissues over time and in space. Therefore, in principle, NDDPs can leverage beneficial outcomes of therapeutics by enhancing drug efficacy, reducing toxicity and required dosage which are considered ideal for anti-cancer drug delivery. Moreover, it is possible to impart imaging functions to NDDPs [26] (cancer can be diagnosed for individualized therapy) and they possess an invaluable ability to respond to a multitude of stimuli (i.e., temperature, pH, chemicals, pressure, and magnetic and electric fields etc.) [27]. Here, in this present review, we summarize recent advances on promising inorganic NDDPs for targeted release of anti-cancer chemotherapeutic agents together with encompassing their therapeutic performance at tumor sites. Our effort highlights the emergent targeted inorganic NDDPs bearing great potential of clinical impacts for future research.

## 2. Inorganic NDDPs Implicated in Anti-Cancer Therapy

A wide range of inorganic agents have been employed for the development of anti-cancer NDDPs with varying sizes (Figure 1), architectures and surface physicochemical properties aiming to target the tumors. Mesoporous silica nanoparticles (MSNs) carbon nanotubes (CNTs), layered double hydroxides (LDHs), superparamagnetic iron oxide nanoparticles (SPIONs) and calcium phosphate nanoparticles (CPNs) have great potential for cancer treatment in various ways. In the following sections, we discuss in-depth therapeutic potential of these NDDPs in treating the various types of cancer.

### 2.1. MSNs based NDDPs Implicated in Anti-Cancer Therapy

MSNs have attracted considerable attention in the field of nanomedicine, and are extensively being used as promising NDDPs for efficient systematic drug delivery due to their unique physiochemical properties. Their major features include: controllable particle size, large surface area, pore volume, ease of surface functionalization, good biocompatibility and ability to protect the housed drugs from degeneration or denaturation. MSNs based NDDPs with tunable pore size are capable of controlling the drug loading/releasing in targeted fashion with higher cellular uptake and without any premature release [28,29,30]. Other advantages of MSNs are their applications in imaging and in gene interference-based therapeutics [31,32]. Furthermore, MSNs have also emerged as promising vesicles for active and passive targeted delivery which can accumulate in tumor tissues via EPR effect [30].

In recent years, several researchers have shown therapeutic benefits in various pre-clinical cancer settings. Mandal et al. showed a delay of leukemia development in lethally irradiated (1200 cGy) C57BL/6 mice by treating with anthracycline doxorubicin (DOX) loaded MSNs (succinic anhydride and anti-B220 antibody functionalized) [33]. This NDDP preferentially killed the cancerous cells (23.2%) and significantly hindered leukemia (over a period of 160 days compared to control) in mice. In a separate study, selective etching strategy based tunable glutathione sensitive hollow MSNs (containing S-S linkage) NDDP loaded with DOX (~9%) was tested against MCF-7 breast cancer cells in vitro. Due to the large surface area, the drug release was remarkably high (58%) with increased cellular uptake (5.2%) and endolysosomal accumulation compartments. Moreover, the NDDP effectively showed cytotoxic killing behavior of up to 51% of MCF-7 cancerous cells after 24 h [34]. A stimuli-responsive DOX loaded triple layered (core shelled MSNs-fluorescein isothiocyanate labeled hyaluronan-switchable zwitterionic surface) accurate anti-cancer drug delivery system has also showed prolonged blood circulation time in an in vitro study. MSNs nanocarriers effectively achieved antibiofouling ability, maintained the enhanced cellular internalization and targeted drug delivery to HeLa tumor cells and exhibited preferred cytotoxicity against cancer development as compared to free DOX [35]. Similarly, DOX loaded thiol modified MSNs (DOX-mMSN) was synthesized by the sol-gel technique for efficient pH dependent delivery of drug to in vitro cultured cancerous cells. This NDDP displayed profound drug loading (48.56%) and faster release (78% at pH 5) which lead to cell cycle arrest, ROS generation and cytotoxicity against the HeLa cells [36]. In another in vitro investigation, a self-controlled fine releasing (46.6%) strategy of Oxaliplatin (Oxa(IV)) from functionalized MSNs has been reported to have high concentrations of accumulation in lung adenocarcinoma cell line A549. Under acidic stimuli conditions, the side effects of Oxa(IV) were reduced for the treatment of lung adenocarcinoma. This simplified model utilized a prodrug approach to avoid the side effects of Oxa(IV) and decreased the multidrug resistance [37].

Osteosarcoma (bone cancer) is a fatal type of cancer and its peak incidences are commonly seen during the adolescent growth spurt [38]. A pH responsive novel DOX loaded and polyacrylic acid/lectin capped MSNs NDDP has been developed for bone cancer treatment using in vitro model of human osteocarcoma cells (CRL-1543). This multifunctional anti-cancer drug loaded NDDP showed killing of almost 100% CRL-1543 cells at a small dose of 2.5 mL^−1^ (8-fold higher cytotoxicity than bare DOX). The nanosystem exhibited noticeable higher internalization degree (~70%) into human osteosarcoma cells by recognizing cell-surface glycans, (i.e., sialic acids) which are overexpressed on cancerous cells. Overall, this strategy lead to opens up new insights in targeted bone cancer therapy [39]. Glioma is another most lethal type of cancer which accounts for majority of deaths in spite of prevailing treatment options and the survival rate among patients is very poor [40]. Thus, the situation demands latest developments in mainstream cancer therapy to cure glioma. Recently, Heggannavar et al. used an advance in vitro blood-brain barrier model of human primary glioblastoma cells (U87 MG). This blood-brain barrier-permeable NDDP consisting of DOX loaded in magnetic MSNs and fabricated with Pluronic F-127 and transferrin (DOX-MNP-MSN-PF-127-Tf) showed sustain and targeted release of anti-cancer drug [41]. Regarding the cytotoxic potential of NDDP, it showed significant IC_50_ value (0.570 μg/mL vs. blank: 121.98 μg/mL) against U87 MG cell line. Further, under magnetic stimuli, NDDP demonstrated an excellent permeability across human brain microvascular endothelial cells, suggesting a facilitated uptake of DOX in U87 MG cells [41]. To treat the human hepatocellular carcinoma using naturally bioactive prodrugs (i.e., curcumin, quercetin, colchicine), AbouAitah et al. investigated nanoformulation-based amine and folic acid conjugated MSNs (KCC-1 and KCC-2). Both NDDPs demonstrated in vitro steady drug release behavior, sustained intracellular release and uptake, cytotoxicity effects and cellular signaling (caspase-3, H_2_O_2_, c-MET and MCL-1) mediated apoptosis in HepG2 and HeLa cells [42]. In some other research, PEGylation and ordered MSNs loaded with mitoxantrone (MTX) and tumor-homing peptides (CREKA, DTPA), respectively, have been proved as effective anti-cancer NDDPs [31,43]. From the discussion and advances presented above, it could be inferred that multifunctional MSN-based NDDPs could serve as potential controlled and targeted platforms for cancer therapy and have huge potential to be translated into clinical implications in future.

### 2.2. CNTs based NDDPs Implicated in Anti-Cancer Therapy

CNTs are essentially carbon atoms which can be used as nanocarriers for delivering of drugs and genes [44,45]. They are made from graphene sheets rolled into a seamless cylinder (Figure 1) that can be open ended or capped, having a high aspect ratio with diameters as small as 1 nm, and length of several micrometers [44]. In recent years, CNTs have emerged as excellent NDDPs owing to their excellent cell penetration aptitude, anisotropic conductivity/semi-conductivity, thermal properties, ultrahigh surface area (maximizes the ability of CNTs to talk with biological matter), hollow interior (provides an enormous cargo-carrying capacity for drug-loading) and their readily functionalized exteriors which permit the tailoring of solubility/biological recognition [45,46]. Based on layers of CNTs, they are generally divided into two categories (Figure 1): single-walled carbon nanotubes (SWCNTs; consist of single layer of cylinder graphene) and multi-walled carbon nanotubes (MWCNTs; contain multiple layers of graphene sheets) [46,47].

Recently, a biocompatible hydroxypropyl β-cyclodextrin grafted SWCNTs NDDP with high loading capacity (8.43%) resulted in vitro targeted delivery of formononetin (FMN, an anti-cancer drug) to MCF-7 and HeLa cancerous cells. The drug release kinetics showed a sustained release and in vitro cytotoxicity assay revealed that anti-tumor activity of CD-SWCNTs-FMN is much more stronger than that of free FMN [48]. CNTs are also well known for ideal NIR photothermal ablation therapy because they increase the temperature within tumors as a function of light intensity and CNT dose-dependent manner [49]. Metformin (MET) is an anti-cancer drug which has direct action on the growth of a wide range of cancers and their proliferation such as breast, colon, glioma, ovarian and endometrial cancers [50,51]. An NIR-responsive MET-loaded MWCNTs system remarkably demonstrated anti-cancer response on the HepG2 cell line at a very low dose of MET relative to only 1/280 of typical doses in monotherapy (35: 10,000–30,000 µM). Mechanistically, heat generated from CNTs upon NIR irradiation (2 W·cm^−2^, 5 min treatment time) resulted in a strong and highly localized hyperthermia-like condition that facilitated the in vitro enhancement of cytotoxic effects of this NDDP against hepatocellular carcinoma (cell viability decreased to 62.2%, *p* < 0.0001) demonstrating a promising use of localized heat generation from CNTs to intensify the efficacy of MET for cancer therapy [51]. Multispectral fluorescence imaging represents significant advancements in cancer drug delivery research. In this perspective, efficiency of programmed thermo-sensitive hydrogel combined chitosan-MWCNTs system loaded with DOX and rhodamine B (RB) as dual drug delivery vehicle was monitored in vivo. Balb/c nude mice were subcutaneously administered with RB-DOX-CNTs/hydrogel and tracked by a multispectral fluorescence imaging system. Among the tested anti-cancer drugs, DOX exerted a significantly slower release rate vs. RB rate from hydrogel, affirming the system may prove a potential NDDP with programmed release of anti-cancer drugs in combined drug administration therapy for treatment of cancer [52]. Karthika et al. (2018) [53] designed MWCNTs TiO_2_-Au to enhance the biocompatibility of NDDP for in vitro DOX delivery. MWCNTs TiO_2_-Au elucidated hemolytic and antimicrobial activities and high antioxidant potential as well as selective pH (5.5)-dependent in vitro drug delivery (releasing capacity of 90.66%) against A549 and MCF7 cancer cell lines [53]. In a separate in vitro study, PEGylated MWCNTs were optimized as a versatile vector to observe the cytotoxicity and DOX-loading capacity for tumor-specific intracellular-triggered release of DOX based on MWCNTs length and degree of PEGylation. PEG-MWCNTs (≤300 nm) showed better cytocompatibility and high drug-loading capacity of 0.55 mg. DOX released rapidly (cumulative release of 57%) under acidic pH media conditions leading to enhanced cancer inhibitory efficiency of this NDDP against HepG2 cells [54]. For antitumor immunotherapy, CNTs also act as antigen-presenting carriers to improve immunogenic tumor-based peptides/antigens to trigger the humeral immune response within the tumors [55,56].

Fullerenes are carbon allotropes with a large spheroidal molecule consisting hollow cage of sixty or more carbon atoms [57]. In particular, fullerenes have been shown to be extremely useful in various biomedical applications such as in nanomedicine and in anti-cancer drug delivery systems [58,59] (Figure 1). Recently, a non-toxic and biocompatible 5-fluorouracil (5-FU) loaded B_24_N_24_ fullerene has potentially been explored for the possible targeted delivery in cancer therapy. In vitro drug release profiles revealed the potential of implication this NDDP under low pH conditions of cancerous cells where the drug delivery cluster is believed to be significantly protonated, thereby improving drug separation from B_24_N_24_ fullerene and release kinetics from surface of the cluster [60]. Fullerene exhibiting amphiphilic and self-assembly characters is of particular interest for sustained and delayed drug release performance, and tumor imaging [61,62]. In this sense a C_60_R_5_Cl fullerene (R: 4-aminobutyric methyl ester or glutamic acid methyl ester or phenylalanine methyl ester; size 80–135 nm depending on R) has been successfully used for in vitro delayed release of several anti-cancer drugs (cyclophosphamide, 5-FU, cisplatin). The study demonstrated that drug loading and release rate depend on the nature of R [62]. Similarly another novel amphiphilic phototheranostic platform based on fullerene C_70_ and Chlorin e6 (Ce6) nanovesicles (FCNVs) also showed effective tumor imaging and cancer treatment potential. FCNVs exhibited high Ce6 loading efficiency (~57 wt%), excellent response in NIR region, enhanced cellular uptake of Ce6 (in vitro and in vivo), good biocompatibility and total clearance out of the body. These unique properties suggest that FCNVs could ideally be applied as an ideal theranostic agent for simultaneous imaging and photodynamic therapy of tumors [61]. In another study, Docetaxel (DTX) was delivered to human breast cancer cells (MDA-MB-231 cell line) with enhanced efficacy and safety using aspartic acid linked fullerenols. Cytotoxic investigations proved that conjugated NDDP has ~4.3-fold lower IC_50_ with significant cellular uptake and high bioavailability of drug (~5.8-fold higher as compared to free DTX) and nano-constructs were also immuno-compatible [63]. DTX has also been delivered in another in vitro cancer model using carboxylated and acylated C_60_-fullerenes as carrier vehicles to MCF-7 and MDA-MB231 cancerous cells. This nano-construct depicted high DTX bioavailability (4.2 times), 50% less drug clearance and more compatibility towards erythrocytes. This strategy could potentially be used to enhance the delivery and efficacy potential of anticancer agents, especially those belonging to BCS class IV [64]. Taken together, it could convincingly be summed up that both CNTs and fullerenes exhibit controlled release, high drug-loading capacity and extended period of blood circulation properties as promising candidates for anti-cancer NDDPs.

### 2.3. LDHs Based NDDPs Implicated in Anti-Cancer Therapy

Known as anionic clays, LDHs (Figure 1) have attracted huge attention of researchers owing to their great potential to be used as anti-cancer NDDPs. They are low cost and have ease of preparation, high drug loading efficacy, full protection for loaded drugs, considerable drug delivery, biocompatibility, anion exchange capability, pH-responsive drug release, efficient cell membrane penetration, good endosomal escape, biodegradation in the cellular cytoplasm (pH between 4 and 6) and above all, the drug release rate can be tuned by changing the interlayer anion [65,66,67]. In addition, the internal/external surfaces of LDHs can easily be functionalized and modified to incorporate the targeting function, and their high specific surface area and better chemical stability make them attractive substances for diverse biomedical applications. Structurally, LDHs are layers of divalent metal ion (i.e., Mg^2+^, Ca^2+^, Ni^2+^, Zn^2+^) with a trivalent metal ion isomorphically substituted to give the layers a net positive charge [67]. Th charge is balanced by interlayer hydrated anions, resulting in a multilayer of alternating host layers with exchangeable gallery anions (i.e., Cl^−^, NO^3−^, CO_3_^2−^).

As effective adjuvants for cancer immunotherapy, multi-target therapeutic vaccines loaded LDHs demonstrated strong dispersion-stable LDH-based vaccine induced cytotoxic T-lymphocyte responses for effective inhibition of melanoma [68]. Recently, Allou et al. and colleagues developed an LDH intercalated hybrid NDDP consisting of carboxymethyl cellulose cross linked citric acid and anti-cancer drug norfloxacin (NOR) [69]. NOR release behaviors were investigated, in vitro, under acidic conditions and NDDP showed non-toxicity against human ovarian cancer (PA-1 cell line), suggesting the potential use of nanohybrids as drug carriers [69]. Another Mg-Al-LDH-MTX-based NDDP has also showed a tremendous potential for anti-cancer drug delivery in vivo [70]. Methotrexate (MTX) possesses some drawbacks regarding its solubility and toxicity; to overcome these issues, a nanoceramic matrix (Mg-Al-LDH-MTX) encapsulated in poly (d,l-lactide-*co*-glycolide) polymer was reported as a safe antitumor NDDP for osteosarcoma in Balb/c nude mice as compared to bare MTX [71]. Consistently, a well dispersible nanocomposite of SiO_2_-Mg-Al-LDH delivered MTX to the human osteosarcoma cells (U2OS) significantly inhibited the U2OS cell growth [70]. In a separate study, MTX intercalated in two-dimensional and colloidally stable nanohybrid was implicated in an in vivo (male Balb/c mice) model of cervical cancer. Results demonstrated that NDDP-incorporated MTX indicated better biodistribution and targeting properties such as tumor-to-blood (5-fold higher) and tumor-to-liver (3.5-fold higher) ratios than free MTX at an interval of 30 min post-injection [72]. Anticancer drug raloxifene hydrochloride when intercalated into a series of Mg-Al-LDHs with varying interlayer exchangeable anions (NO^3−^, CO_3_^2−^ and PO_4_^3−^) has been reported to release the drug in a controlled manner [67]. A rapid and sustained drug release rate was facilitated by nitrate-bound LDH-drug vs. loose phosphate-bound LDH-drug in vitro (HeLa cervical cancer cell line, murine breast cancer cell line 4T1) and in Balb/c mice model of cancer.

Etoposide (VP16) is an important anti-cancer drug for various cancers but at the same time it possesses some side effects including frequent hair loss, leukopenia and thrombocytopenia. In order to overcome the side effects associated with VP16, Zhu et al. developed a pH-responsive LDH-VP16 nanohybrid NDDP for in vitro lung cancer model which induced 2.3-fold apoptosis in the A549 cell line targeting mitochondrial and stocking cells in G1 phase [73]. An in vivo study on Balb/c mice resulted in prolonged blood circulation half time of LDH-VP16 (from 6.68 to 98.78 h), inhibited the tumor growth up to 60.5% and strongly reduced the hematotoxicity and hepatic toxicity. Mechanistically, in vitro, LDH-VP16 suppressed the PI3K–AKT signaling pathway to inhibit the tumor growth whereas in xenografts (Ncr nude mice bearing A549) LDH-VP16 stifled the ERBB signaling, leading to increased anti-cancer efficacy [73]. Positively charged LDH can easily penetrate into negatively charged cell membranes through the clathrin-mediated endocytosis pathway. Taking advantage of this, Li et al. [74] employed a combinatorial strategy using LDH to simultaneously deliver the 5-FU and Allstars Cell Death siRNA (CD-siRNA) to various cancer cells lines (HCT-116, MCF-7, U2OS,) which resulted in significantly higher cytotoxicity against cancerous cells than single treatment either with 5-FU or CD-siRNA. Recently, sheet-like mannose functionalized SiO_2_-coated LDH (Mn-SiO_2_-LDH) NDDP has also been effectively used for in vitro CD-siRNA-based anti-cancer gene therapy to kill osteosarcoma (U2OS) cancerous cells [75]. Apart from pharmaceutical drugs, natural bioactive anti-cancer molecules are also gaining high interest in recent research [76,77]. Curcumin (CUR) loaded LDH (~22% loading efficiency) significantly reduced in vitro cell migration and invasion of glioblastoma on A172 cell line by downregulating the PI3K/AKT/mTOR signaling pathway [78]. Furthermore, gallic acid intercalated into LDH has also been therapeutically exploited (in vitro) against adenocarcinoma resulting reduction of the cancerous cell viability by 67% [79]. In conclusion, the uniqueness of LDHs structure has highlighted their use for future clinical cancer research owing to their capability of intercalating various anti-cancer drugs for sustained delivery and high anti-cancer therapeutic efficiency.

### 2.4. SPIONs Based NDDPs Implicated in Anti-Cancer Therapy

SPIONs consist of an inner magnetic core (i.e., magnetite, Fe_3_O_4_, Ni, Co, maghemite, γ-Fe_2_O_3_) and a coating of hydrophilic organic polymers (polysaccharides, dextran, alginate, poly(ethylene glycol), poly(vinyl alcohol) (Figure 1). In recent research, both in vivo and in vitro, they are gaining high importance to be used in targeted chemotherapy, in magnetic resonance imaging (MRI) and in transfection due to their intrinsic properties. SPIONs possess inherited magnetism and they are nontoxic, external stimuli responsive, biodegradable, biocompatible, visualized by MRI, capable of inducing local hyperthermia in tumor regions and could efficiently be cleared from the human body via iron metabolism pathways [80,81]. The outer coating of biocompatible polymers helps to shield the magnetic particles from the surrounding environment and can also be functionalized by targeting ligands (avidin, biotin, carboxyl groups, carbodiimide etc.) which, in turn, increase the targeting yield by acting as anchoring points for the coupling of cytotoxic drugs or target antibodies to SPIONs. Furthermore, these magnetic drug-bearing nanocarriers are also highly responsive to external magnetic field, which, when locally applied to the target tumor sites results in improved accumulation and biodistribution of SPIONs loaded anti-cancer drug(s) to the desired target tumor tissues. Several methods of SPIONs preparation and functionalization for anti-cancer application have been reported [82,83]. Recently, Kheirkhah et al. used a novel magnetic drug targeting technique (MDT) for the targeted in vivo delivery of DOX to high-grade intramedullary spinal cord tumor in immunodeficient athymic nude Crl:NIH-*Foxn1^rnu^* rats. SPIONs carried DOX successfully localized to tumor sites demonstrating proof-of-concept that MDT may prove an effective and concentrated delivery system without toxicity for systemic administration [84]. In separate work, Foglia et al. synthesized biocompatible ultrafine 3 nm water dispersible silica capped SPIONs by employing one-pot approach. Functionalized with fluorescein isothiocyanate, SPIONs were tested against human colon cancer in vitro (CaCo-2 cell line) and did not interfere with the expression of differentiation markers pro-inflammatory cytokines, suggesting their safe therapeutic biomedical applications [85]. In another in vitro model of hepatocellular carcinoma (HepG2.2.15 cell line), a multi-functional vehicle comprising of methoxy-poly(ethylene glycol)-b-oligo(ε-caprolactone) encapsulated quercetin and SPIONs micelles also showed increased cytotoxicity against hepatitis B virus-transfected liver cancer and caused cell cycle arrest at G_0_/G1 phase leading to potent inhibition of cancer growth [86]. To overcome the chemo-resistance in prostate cancer, a novel formulation of DTX loaded prostate specific membrane antigen SPION (J591-SPION-DTX) exhibited potent in vitro anti-cancer efficacy by modulating several molecular processes (i.e., inducing apoptosis associated proteins, downregulation of anti-apoptotic proteins). The cellular uptake of J591-SPION-DTX by C4-2 (PSMA^+^) cells was remarkably high, suggesting the tumor specific targeting of this NDDP for targeted prostate cancer therapy [87]. SPIONs also have the potential to cure cancer by generating local heat when exposed to an alternating magnetic field. It is has been established previously that cancer cells are susceptible to hyperthermia (temperature increases to ∼43 °C for 30–60 min) which triggers apoptosis [88,89]. Taking advantage of this fact, magnetic droplet vaporization (MDV) strategy has recently been utilized in the development of alternative current generated magnetic field responsive and ultrasound imaging Perfluorohexane encapsulated SPIONs (PFH-HION). This NDDP efficiently raised the temperature of tumor tissues (102.3 ± 9.8 °C in 3 min), produced microbubbles of PFH and led to ablation and complete removal of cancer in nude mice bearing MDA-MB-231 xenograft of human breast tumors [90]. This is a unique development in targeted cancer drug delivery approach with high versatility/performance which could to promote the clinical translations of intelligent diagnostic and therapeutic modalities for battling the cancer. However, hyperthermia alone has not been recommended for cancer treatment and it is often used as an adjuvant to other forms of therapy such as surgery, radiotherapy and chemotherapy. Concluding with our discussion, there is a high hope that future cancer research will focus more on combining chemotherapy and hyperthermia using multifunctional SPIONs.

### 2.5. CPNs based NDDPs Implicated in Anti-Cancer Therapy

CPNs (Figure 1) have commendable properties such as colloidal stability, biocompatibility, bioactivity, tunable biodegradability, and they can encapsulate negatively charged chemotherapeutic agents by chelating calcium ions during formation of CP nanocrystals. The dissolution rate of CP could be contained by adjusting its crystallinity, thereby promoting the development of efficient drug and genes delivery nanosystems [91,92,93]. Recently, DOX and MRI contrasting agent-loaded multifunctional CPN-based NDDP (A54-CaP/Gd-DTPA/DOX) showed promising outcomes in treatment of hepatic tumors with real-time monitoring properties via T1-weighted MRI. Under acidic conditions and by A54 binding, the in vitro internalization behavior of NDDP was 1.9-fold faster in BEL-7402 cells, whereas, in vivo investigations revealed that A54-CaP/Gd-DTPA/DOX inhibited the tumor to great extent (95.38% antitumor efficacy) owing to higher distribution and longer retention time in tumor tissues [94]. Liu et al. (2018) also beneficially exploited Triptolide and CUR-loaded CPN nanosystems in an animal model of ovarian cancer, suggesting the maximum synergistic cancer killing effects of the two drugs as high as 68.78% [95]. The developments in the field of immune-oncology, via enhancing a host’s immune system to fight the cancer, are also gaining high importance in contrast to conventional cancer therapies [96]. In this perspective, an anti-cancer immunosuppressive agent (FTY720) co-delivered with Beclin 1 siRNA by CPNs successfully induced the protective autophagy and promoted in vitro apoptosis in hepatocellular carcinoma (SMMC-7721 and A549 cell lines). These new insights in treatment of liver cancer, based on co-delivered small molecules and siRNA, could potentially be translated into cancer clinical trials [97].

Lipid CPNs have been found to achieve both systemic delivery of drugs/genes to the lymphatic system and imaging of lymph node metastasis [98]. Tang et al. developed a lipid coated CPNs and folic acid and/EGFR-specific single chain fragment antibody conjugated dual drug/gene nanosystem (LCP-50FA-75scFv NPs) for target delivery and improved cellular uptake of chemotherapeutic agents in an experimental breast tumor model of MDA-MB-468 nude mice [99]. In a separate study, a lipid-coated hollow CPNs system (LCP) was also tested, in vitro, for combined delivery of two chemotherapeutic drugs (DOX, PTX) to human lung cancer A549 cells. The combined effects resulted in sustained in vitro release of drugs with better biocompatibility, cellular uptake of LCP and less cytotoxicity [100]. Furthermore, a novel dual pH-responsive DOX loaded lipid-bilayer-coated polyacrylic acid/CPNs nanosystem exhibited excellent biocompatibility and significantly increased the cellular accumulation of DOX in HepG2 cells [101]. PEGylated CPNs hybrid micelles are also considered to enhance the in vivo accumulation of siRNA in tumors and promote their gene-silencing activity [102]. A novel co-delivering CP-polymer hybrid NDDP was reported to effectively deliver the different chemotherapeutic agents (i.e., microRNA-221 and microRNA-222 inhibitors) and PTX for the in vitro treatment of triple-negative breast cancer (MDA-MB-231 cell line) [103]. Taken together, CPNs can be applied as emerging NDDPs for their clinical translation.

## 3. Conclusions and Future Outlook

Inorganic NDDPs have been proved as potential contenders in cancer therapy replacing the drawbacks of conventional chemotherapy. Recent research reported the high anti-cancer drug(s) loading/releasing profiles of inorganic NDDPs, effective/targeted delivery of chemotherapeutic agents loaded in inorganic NDDPs, reduced toxicities for healthy cells and cancer improving outcomes of using inorganic NDDPs in a variety of in vitro/in vivo cancer models. Thus, in future, it could be stipulated that unique attributes of inorganic NDDPS may allow clinicians to recognize these emerging anti-cancer candidates as new treatments (monotherapy of cancer) or as adjuncts to existing treatment options (combined therapy of cancer) in order to achieve the overall therapeutic effectiveness in nano-oncology. Apart from major advances made in inorganic NDDPs for treatment of experimental cancers, much work lies ahead to monitor their effectiveness in clinical trials as most of these NDDPs have been tested in small animal models (or in vitro), achieving good therapeutic results. This could spark a quest in future research, leading to translation of animal results into clinical success. We are anticipating a bright future for cancer treatment in coming decades or at least with safer and more efficient cancer treatments approaches, ensuring proper drug localization at the site of action in a controlled manner.

## Figures and Tables

**Table 1:** Recent experimental (in vitro and in vivo) investigations highlighting the therapeutic potential of inorganic NDDPs for treatment of various cancers.

NDDP	Inorganic Agent(s)	Ther. Agent	DLC/DRE (%)	Stimuli	Study Model	Cancer Type	Preventive Outcomes of NDDPs in Cancer
nGO-DOX-cPEG [6]	Graphene	DOX	~70/>60	NIR, pH	In vitro: PC3, DU145, LNCaP In vivo: BALB/c nude	Prostate	In vitro: ↑ cancer cells killing by ~80%, ↑ cellular drug uptake, ↑ apoptotic effects (higher expression of p53, p21, bax, c-caspase 3). ↓ cytotoxicity for normal cells. In vivo: ↑ site selective accumulation and prolong blood circulation (>24 h), ↑ sustained drug release and retention. ↓ tumor growth and volume.
HSG-DOX [7]	Graphene	DOX	49.2/>50	NIR, Redox	In vitro: MDA-MB-231 In vivo: BALB/c nude	Breast	In vitro: ↑ targeted delivery to HA-receptor over expressing tumors, ↑ endo/lysosomal escape, ↑ cytoplasmic release. ↓ cytotoxic even at <10 µg mL^−1^. In vivo: ↑ preferential accumulation (36 h) and tumor targeting, ↑ sustained drug release and retention. ↓ tumor growth and volume, ↓ side effects (evidenced by improvements in body weight).
GO-CHI-HA-SNX-2112 [8]	Graphene	SNX-2112	>110/>40	pH	In vitro: A549, NHBE In vivo: Sprague-Dawley	Lung	In vitro: ↑ CD44 receptor mediated cellular uptake and intracellular drug distribution, ↑ cell apoptosis as high as 36.59%, ↑ blood compatibility (haemolysis analysis). ↓ cytotoxic (evidenced by cell viability). In vivo: ↑ immune-tolerant, ↓ tissue swelling, inflammation and lesion formation, ↓ necrocytosis and fibrous proliferation, ↓ systemic side effects.
DOX-FA-rGO/ZnS:Mn [9]	Graphene, Zinc, Manganese	DOX	~35/95	pH	In vitro: MDA-MB-231, NIH-3T3 In vivo: NS	Breast	In vitro: ↓ cancer cells viability by 50%, FA functionalization decreased the toxic response up to 72 h, FA functionalized system further improved the drug disperseability, bioavailability and selective drug release. In vivo: NS
H-MnO_2_-PEG-C&D [10]	Manganese	Ce6, DOX	>86/>90	pH, NIR	In vitro: 4T1 In vivo: Balb/c mice	Breast	In vitro: relieve tumor hypoxia, ↓ high photo-toxicity for cancer cells, ↑ intracellular accumulation, ↑ killing cancer. In vivo: ↑ efficient tumor accumulation, ↑ decomposition of H_2_O_2_ into O_2_ and accumulation tumor, ↓ reduce tumor hypoxia, ↓ tumor size, ↓ side effects (evidenced by improvements in body weight), ↑ macrophages infiltration, ↑ cytotoxic T lymphocytes, ↑ TNF-α secretion
DOX-PVP-AuNPs [11]	Gold	DOX	NR/91	pH	In vitro: 549, H460, H540 In vivo: NS	Lung	In vitro: ↑ inhibition of lung cancer cells proliferation, ↑ ROS generation, ↑ cancer cell apoptosis, ↑ mitochondrial membrane depolarization potential (more cellular uptake), ↑ expression of apoptotic cytochrome oxidase C and bax, ↑ expression of tumor suppressing p21, p53, ↑ expression of caspases 3 and 9. In vivo: NS
BLM-DOX-PEG-AuNPs [12]	Gold	BLM, DOX	33/2.3	pH	In vitro: HeLa In vivo: NS	Ovarian	In vitro: ↑ cytotoxicity for cancerous cells at lower concentration, ↑ active targeting, ↑ cancer cell apoptosis, ↑EC50 values. ↓ agglomeration. In vivo: NS
Se-Au-mSiO_2_-DOX [13]	Gold, Selenium, Silicon	DOX	8.1/80	Laser NIR	In vitro: MCF-7, MDA-MB-231 In vivo: BALB/c nude	Breast	In vitro: ↑ reversing multidrug resistance strategy, ↑ intracellular drug release in lysosomes, ↑ cytotoxic effect and cancer cells killing (evidenced by cell shrinkage, chromatin condensation, degeneration, nuclear fragmentation), ↑ apoptosis (~40% of cells in late apoptosis), ↑ ROS generated mediated mitochondrial dysfunction, ↑ expression of tumor suppressing p21, p53, ↑ Bid and Bad expression. ↓ GSH/GSSG ratio, ↓ PARP expression, ↓ Bcl-xl expression, ↓ Src/FAK/AKT pathways. In vivo: ↑ nanoparticles accumulation in tumor sites (24 h), ↑ cellular apoptosis by 10 fold, ↑ caspase 3 by 11 fold, ↑ PARP by 13 fold. ↓ tumor progression, ↓ tumor volume by 5 fold, ↓ adverse effects.
Ag-GQDs-DOX [14]	Silver	DOX	NR/NR	pH	In vitro: DU145, HeLa In vivo: NS	Prostate, Cervical	In vitro: ↑ antitumor activity, ↑ apoptosis in cancer cells, ↓ affecting the viability of normal cells, ↑ caspase-3 activity, ↑ caspase-7 activity, ↑ cellular drug uptake. In vivo: NS
CPT-CEF [15]	Iron	CPT	NR/65	pH, Temp.	In vitro: HT29, A549 In vivo: NS	Colon, Lung	In vitro: ↓ cancer cells viability, ↑ membrane rupturing and nuclear fragmentation, ↑ induction of cell toxicity cancer cells, ↑ early apoptosis, ↓ cancer cells proliferation, ↑ mitochondrial membrane depolarization, ↑ caspase-3 activity. In vivo: NS
HA-FeOOH-PPy NRs [16]	Iron	HA	NR/NR	Laser	In vitro: MDA-MB-231 In vivo: BALB/c nude	Breast	In vitro: ↓ cancer cells viability, ↑ quick cellular uptake, ↑ selectivity toward CD44 expressing cancer cells, ↑ biocompatible, ↑ cancer cells killing efficiency, ↑ localized drug distribution around the tumor. In vivo: ↑ tumor volume growth (by 11 times), ↓ side effects (evidenced by improvements in body weight).
MNP-HC [17]	Iron	TZ	NR/NR	NS	In vitro: SKBR3, MDA-MB-231 In vivo: NS	Breast	In vitro: ↑ up to 97% binding to the tested cell lines, ↑ site-specific phosphorylation in catalytic domain of HER2, ↑ cellular uptake, ↑ anti-cancer proliferative effect (antibody dependent cell mediated cytotoxicity) by 41.8%, ↑ p27kip1 expression. In vivo: NS
DOX-MGNSs [18]	Iron, Gold, Silica	DOX	65.8/86.2	pH, Temp.	In vitro: ATCC CCL-2 In vivo: NS	Ovarian	In vitro: ↑ cytoxicity for cancer cells, ↑ cellular uptake, ↑ anti-cancer proliferative effect, ↑ disruption of ruffles in cells (loss of morphology). In vivo: NS
HA-ionic-TPP-DOX [19]	Phosphonium	DOX	31.4/91	pH	In vitro: MCF-7/ADR In vivo: BALB/c nude, Tg(fli1a:eGFP)^+/−^	Breast	In vitro: ↑ cellular uptake and cytoxicity in tumor cells, ↑ prolong blood circulation, ↑ drug distribution in tumors, ↑ enhanced permeability and retention, punctate distribution (selectively mitochondrial accumulation), ↑ ROS generation. In vivo: ↑ tumor targeting, ↓ tumor growth and tumor progression, ↓ tumor volume, ↓ adverse effects (cardiotoxicity).
DOX-BP-Hyd [20]	Phosphorus	DOX	NR/38.8	NIR, Light	In vitro: MDA-MB-231, A549, HeLa, B16 In vivo: BALB/c nude	Breast, Lung, Cervical, Melanoma	In vitro: ↑ cell killing ability, ↑ tumor ablation effect, ↑ biodegradability, ↑ biosafety. In vivo: ↑ localized drug distribution around the tumor site, ↑ sustained release (<12 h), ↓ tumor growth and volume, ↓ acute side effects (evidenced by improvements in body weight), ↓ toxic to normal tissues.

NDDPs: nano-drug delivery platforms; Ther.: therapeutic; DLC/DRE: drug loading capacity and drug release efficiency; ↑: increased/improved/higher/more/upregulate; ↓: reduced/decreased/lower/less/downregulate; DOX: doxorubicin; nGO-DOX-cPEG: DOX incorporated lateral nanodimentional graphene oxide chitosan polyethylene glycol flakes; NIR: near-infrared; p21: cyclin dependent inhibitor kinase 1; p53: tumor protein 52; bax: BCL2 associated X; h: hours; HSG-DOX: DOX incorporated in bioreducible hyaluronic acid graphene oxide nanosheets; GO-CHI-HA-SNX-2112: SNX-122 incorporated in chitosan modified graphene oxide nanocomposites; NHBEs: normal human bronchial epithelial cells; DOX-FA-rGO/ZnS:Mn: DOX incorporated in reduced graphene oxide manganese doped zinc sulfide quantum dots functionalized with folic acid functionalized; NS: not studied; FA: folic acid; H-MnO_2_-PEG-C&D: Ce6 and DOX incorporated in hollow manganese oxide nanoshells functionalized polyethylene glycol; Ce6: chlorine e6; DOX-PVP-AuNPs: DOX incorporated in polyvinylpyrrolidone stabilized gold nanoparticles; NR: not reported; ROS: reactive oxygen species; BLM-DOX-PEG-AuNPs: BLM and DOX incorporated in polyethylene glycolated gold nanoparticles; BLM: bleomycin; Se-Au-mSiO_2_-DOX: DOX incorporated in nanoselenium over coated mesoporous silica capped gold nanorods; GSH/GSSG: glutathione/oxidized glutathione; PARP: poly ADP ribose polymerase; Bcl-xl: B-cell lymphoma extra-large; Src/FAK: steroid receptor coactivator/focal adhesion kinase; Ag-GQDs-DOX: DOX incorporated in PEGylated silver nanoparticles decorated with graphene quantum dots; CPT-CEF: CPT incorporated in a composite nanoparticle of magnetic iron oxide (Fe_3_O_4_) and β-cyclodextrin cross-linked with ethylenediaminetetraacetic acid (EDTA); CPT: Camptothecin; HA-FeOOH-PPy NRs: hyaluronan coated FeOOH@polypyrrole nanorods; HA: hyaluronan; HA-ionic-TPP-DOX: DOX incorporated in triphenylphosphonium (linked to hyaluronic acid by ionic bond) supra-molecular self-assembled structures; Tg(fli1a:eGFP)^+/−^: Transgenic zebrafish; DOX-BP-Hyd: DOX incorporated in nanocomposite of black phosphorus nanosheets and hydrogel; MNP-HC: half chain trastuzumab incorporated in magnetic iron oxide nanoparticles (MNP-HC); TZ: trastuzumab; HER2: human epidermal growth factor receptor 2; p27Kip1: cyclin-dependent kinase inhibitor p27Kip1; DOX-MGNSs: DOX incorporated in ma/gnetic and gold nanoparticles embedded silica nanoshuttles; Temp: temperature.
